# Ehrlichia chaffeensis EplA Interaction With Host Cell Protein Disulfide Isomerase Promotes Infection

**DOI:** 10.3389/fcimb.2020.00500

**Published:** 2020-09-23

**Authors:** Ryan S. Green, Jerilyn R. Izac, Waheeda A. Naimi, Nathaniel O'Bier, Edward B. Breitschwerdt, Richard T. Marconi, Jason A. Carlyon

**Affiliations:** ^1^Department of Microbiology and Immunology, Virginia Commonwealth University Medical Center, School of Medicine, Richmond, VA, United States; ^2^Department of Clinical Sciences and the Intracellular Pathogens Research Laboratory, Comparative Medicine Institute, College of Veterinary Medicine, North Carolina State University, Raleigh, NC, United States

**Keywords:** *Ehrlichia*, protein disulfide isomerase, Intracellular bacteria, bacterial invasion, adhesin, anaplamataceae

## Abstract

*Ehrlichia chaffeensis* is an obligate intracellular bacterium that invades monocytes to cause the emerging and potentially severe disease, monocytic ehrlichiosis. Ehrlichial invasion of host cells, a process that is essential for the bacterium's survival and pathogenesis, is incompletely understood. In this study, we identified ECH_0377, henceforth designated as EplA (*E. chaffeensis* PDI ligand A) as an *E. chaffeensis* adhesin that interacts with host cell protein disulfide isomerase (PDI) to mediate bacterial entry into host cells. EplA is an outer membrane protein that *E. chaffeensis* expresses during growth in THP-1 monocytic cells. Canine sera confirmed to be positive for exposure to *Ehrlichia* spp. recognized recombinant EplA, indicating that it is expressed during infection *in vivo*. EplA antiserum inhibited the bacterium's ability to infect monocytic cells. The EplA-PDI interaction was confirmed via co-immunoprecipitation. Treating host cell surfaces with antibodies that inhibit PDI and/or thioredoxin-1 thiol reductase activity impaired *E. chaffeensis* infection. Chemical reduction of host cell surfaces, but not bacterial surfaces with tris(2-carboxyethyl)phosphine (TCEP) restored ehrlichial infectivity in the presence of the PDI-neutralizing antibody. Antisera specific for EplA C-terminal residues 95-104 (EplA_95−104_) or outer membrane protein A amino acids 53-68 (OmpA_53−68_) reduced *E. chaffeensis* infection of THP-1 cells. Notably, TCEP rescued ehrlichial infectivity of bacteria that had been treated with anti-EplA_95−104_, but not anti-EcOmpA_53−68_. These results demonstrate that EplA contributes to *E. chaffeensis* infection of monocytic cells by engaging PDI and exploiting the enzyme's reduction of host cell surface disulfide bonds in an EplA C-terminus-dependent manner and identify EplA_95−104_ and EcOmpA_53−68_ as novel ehrlichial receptor binding domains.

## Introduction

Human monocytic ehrlichiosis (HME) is a potentially fatal tick-borne disease first identified in Fort Chaffee, Arkansas in 1986 (Maeda et al., 1987; Anderson et al., 1991; Ismail and Mcbride, 2017). From 2000 to 2017, the number of reported cases in the United States increased by more than 800%. The incidence of HME is greatest in the Southeast and South-central United States (Centers for Disease Control and Prevention, 2020) where in 2017 the reported incidence of the disease was estimated at ~6 cases per million persons. This figure is considered to be an underestimate due to non-compliance with reporting and misdiagnosis. Prospective studies suggest that the incidence of HME in endemic areas may be as high as 100–200 cases per million (Olano et al., 2003; Walker, 2005; Hidalgo et al., 2009; Ismail and Mcbride, 2017). Although the majority of HME cases are reported in the U.S., cases have also been documented in Europe, Asia, South America, and Africa (Walker and Dumler, 1996; Paddock and Childs, 2003). HME presents as an acute non-specific febrile illness. Laboratory abnormalities may include leukopenia, thrombocytopenia, anemia, and elevated serum hepatic transaminases. If diagnosed and treated early, HME typically resolves without complication. In patients that receive delayed or no treatment, complications can develop including meningoencephalitis, respiratory distress syndrome, acute renal failure, and hypotensive shock. HME patients are also susceptible to opportunistic fungal and viral infections. Approximately 50–60% of patients with severe disease require hospitalization. In immunocompromised individuals, HME can be fatal (Ismail and Mcbride, 2017).

The causative agent of HME is *Ehrlichia chaffeensis*, an obligate intracellular bacterium in the *Anaplasmataceae* family that is maintained in nature in a zoonotic cycle between ticks and persistently infected hosts such as white-tailed deer and canids. It is vectored primarily by *Amblyomma americanum*, although other *Amblyomma* spp. and other genera of ticks may also contribute to disease transmission (Starkey et al., 2013; Ismail and Mcbride, 2017). *E. chaffeensis* progresses through a biphasic infection cycle similar to that of other vacuole-adapted obligate intracellular bacteria including other *Ehrlichia* spp., *Anaplasma* spp., *Chlamydia* spp., and *Coxiella burnetii* (Kocan et al., 1984, 1990; Heinzen et al., 1999; Zhang et al., 2007; Troese and Carlyon, 2009; Fischer and Rudel, 2018). The infectious dense-cored (DC) form enters host cells via pathogen-orchestrated receptor-mediated uptake to reside within a host cell-derived vacuole that avoids lysosomal fusion. The DC transitions to the non-infectious reticulate cell (RC) form that divides by binary fission. RCs convert to DCs that subsequently exit to reinitiate the infection cycle (Zhang et al., 2007). While some *E. chaffeensis* adhesins and host cell receptors have been discerned (Popov et al., 2000; Cheng et al., 2011; Mohan Kumar et al., 2013, 2015), disrupting these interactions fails to ablate infection. Thus, the full complement of adhesin-receptor pairs and how they mechanistically drive ehrlichial cellular entry into monocytic cells are incompletely defined.

Protein disulfide isomerase (PDI), a member of the thioredoxin superfamily of redox proteins, is emerging as a commonly-utilized receptor for infection by intracellular pathogens. PDI is expressed in nearly all mammalian cell types and performs thiol-disulfide oxidoreductase, disulfide isomerase, and redox-dependent chaperone activities. It is enriched in the endoplasmic reticulum, but is also found in the nucleus, cytoplasm, and at the cell surface (Ali Khan and Mutus, 2014). PDI at the cell surface functions exclusively as a thiol reductase (Jiang et al., 1999; Zai et al., 1999; Gallina et al., 2002), and this activity is important for internalization into host cells by HIV, Dengue virus, *Leishmania chagasi, Chlamydia trachomatis*, and another *Anaplasmataceae* member, *Anaplasma phagocytophilum* (Barbouche et al., 2003; Ou and Silver, 2006; Abromaitis and Stephens, 2009; Santos et al., 2009; Reiser et al., 2012; Stantchev et al., 2012; Wan et al., 2012; Diwaker et al., 2015; Green et al., 2020). The *A. phagocytophilum* adhesin, Asp14 (14-kDa *A. phagocytophilum* surface protein) engages PDI on myeloid cell surfaces to bring the pathogen in sufficient proximity to the enzyme such that it reduces bacterial surface disulfide bridges as a critical step in infection (Green et al., 2020).

Here, we report that the *E. chaffeensis* Asp14 ortholog, ECH_0377, hereafter designated as EplA (*E. chaffeensis* PDI ligand A), interacts with PDI to enable pathogen entry into monocytic cells. Thiol reduction of the host but not ehrlichial surface benefits infection, indicating that bacterial entry mechanisms promoted by EplA and Asp14 interactions with PDI are unique from each other. Antisera specific for the EplA C-terminus significantly inhibits *E. chaffeensis* of THP-1 cells. These data identify EplA as an *E. chaffeensis* adhesin, define how it facilitates cellular invasion, and delineate its functional domain.

## Results

### EplA, an Ortholog of *A. phagocytophilum* Asp14, Is a Surface-Localized Protein that *E. chaffeensis* Expresses During Infection of Monocytic Cells and in *Ehrlichia* spp.-Infected Dogs

**Figure 1:**
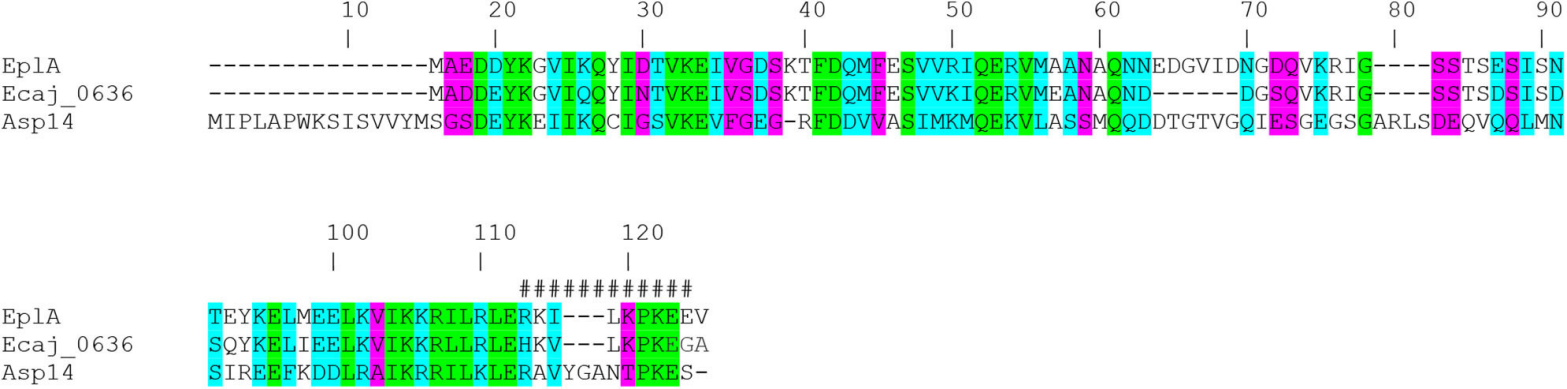
Alignment of *E. chaffeensis* EplA with its orthologs *E. canis* Jake str. Ecaj_0636 and *A. phagocytophilum* Asp14. Asp14 amino acid numbers are listed above the alignment because it is the largest of the three proteins. Green highlighting denotes identical amino acids as defined by Clustal Omega. Turquoise highlighting demarcates highly similar amino acids. Magenta highlighting signifies amino acids that are weakly similar. Hash tags (#) denote the previously confirmed Asp14_113−124_ PDI binding domain.

**Figure 2:**
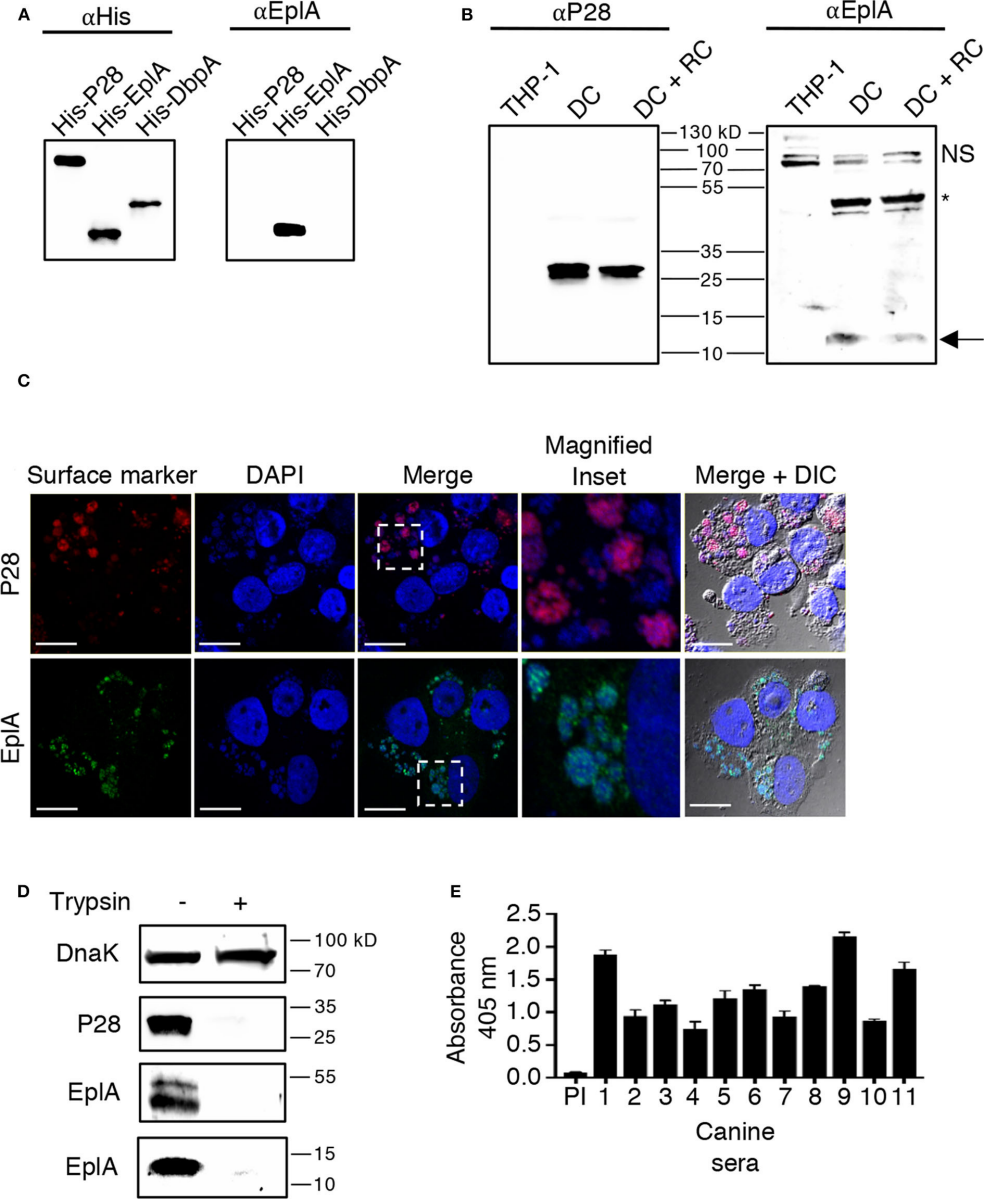
EplA is expressed during *E. chaffeensis* infection of monocytic cells *in vitro* and during natural ehrlichial *in vivo* infection. **(A)** Confirmation of EplA antiserum specificity. Antibody against the 6X-His tag (αHis) or rat antiserum raised against His-tagged EplA (αEplA) was used to screen Western-blotted nickel affinity-purified His-P28, His-EplA, and His-DbpA. **(B)** *E. chaffeensis* expresses EplA during infection of monocytic cells. Antisera raised against His-P28 or His-EplA was used to screen Western blotted-lysates of uninfected THP-1 cells or lysates obtained following either sonication or syringe passage of infected THP-1 cells, which enriches for the DC morphotype or both DC and RC bacteria, respectively. The arrow denotes an *E. chaffeensis*-specific band having an apparent molecular weight that is approximately that expected for EplA. The asterisk indicates an *E. chaffeensis*-specific doublet detected by αEplA that is presumably an EplA multimeric complex or a heteromeric complex containing EplA. NS, host cell doublet that is non-specifically recognized by αEplA. **(C)** EplA exhibits an OMP-like immunolabeling pattern. *E. chaffeensis* infected THP-1 cells were fixed and viewed by indirect immunofluorescence confocal microscopy to determine immunoreactivity with anti-P28 or anti-EplA. Host cell nuclei and bacterial nucleoids were stained with DAPI (4′,6-diamidino-2-phenylindole). The region demarcated by a hatched line box has been enlarged in the Magnified Inset panel. Note that immunolabeling of EplA yielded a punctate pattern on the periphery of DAPI-stained intracellular bacteria that is comparable to that observed for P28, a known *E. chaffeensis* surface protein. Scale bars, 13 μm. **(D)** EplA is exposed on the *E. chaffeensis* surface. *E. chaffeensis* DC organisms were incubated with trypsin or vehicle control, solubilized, and Western-blotted. Immunoblots were probed with antiserum specific for DnaK, P28, and EplA. Results are shown for the anti-EplA immunoreactive bands that correspond to the EplA monomer and EplA-containing multimeric complex as delineated in **(B) (E)** EplA is recognized by sera from *Ehrlichia* spp. infected dogs. His-EplA was screened by ELISA using preimmune sera (PI) or sera from 12 client-owned dogs that had been naturally infected by *Ehrlichia* spp.

EplA is predicted to be a 12.0-kDa protein that is 104 amino acids in length (Hotopp et al., 2006). Figure 1 presents an alignment of EplA and its homologs, Asp14 and *Ehrlichia canis* Jake str. Ecaj_0636. EplA exhibits 28.2% identity and 66.9% similarity to Asp14 and 76.7% identity and 92.3% similarity to Ecaj_0636. EplA residues 95-104 and Ecaj_0636 amino acids 89-98 align with Asp14 residues 113-124 (Asp14_113−124_) that constitute the adhesin's PDI binding domain (Green et al., 2020). Because of the importance of Asp14 to *A. phagocytophilum* infectivity (Kahlon et al., 2013; Green et al., 2020), the relevance of EplA to *E. chaffeensis* pathogenesis was examined. As a first step, His-tagged EplA was expressed in *E. coli*, purified, and used as an immunogen to generate rat polyclonal antiserum, the specificity of which was confirmed via Western blot analysis. Anti-EplA recognized His-EplA, but not His-tagged versions of the *E. chaffeensis* P28 outer membrane protein (OMP) or *Borreliella burgdorferi* decorin binding protein A (Figure 2A). Anti-EplA detected bands with apparent molecular weights of 13.4, 47.8, and 48.5 kDa in Western-blotted lysates of *E. chaffeensis*, but not uninfected THP-1 cells (Figure 2B). The larger bands detected by EplA antiserum could be multimeric complexes that contain EplA. Consistent with this possibility, Asp14 migrates as both a 14.0-kDa band that is consistent with its predicted molecular weight and as part of a multimeric complex even when resolved under denaturing conditions (Kahlon et al., 2013). Anti-EplA also detected two high molecular weight bands in *E. chaffeensis* and uninfected THP-1 lysates, which presumably correspond to host proteins that were recovered with the bacterial proteins following homogenization and non-specifically detected by the antiserum. As a control, P28 antiserum detected only bands in *E. chaffeensis* lysates. Anti-EplA immunolabeling of intracellular *E. chaffeensis* in THP-1 cells yielded a punctate pattern on the periphery of intracellular bacteria that was similar to that achieved using P28 antiserum (Figure 2C).

EplA has 12 lysine and six arginine residues distributed throughout its sequence (Figure 1A), making is susceptible to trypsin digestion. If EplA or domains thereof are present on the ehrlichial surface, then exposure of intact DC organisms to trypsin should proteolytically cleave EplA at these sites and consequently reduce one's ability to detect it by immunoblot. This method has been used to validate surface localization of multiple *A. phagocytophilum* and *Chlamydia trachomatis* proteins (Wang et al., 2006; Ojogun et al., 2012; Kahlon et al., 2013; Seidman et al., 2014). Trypsin-treated DC bacteria were solubilized, Western-blotted, and probed with antisera against EplA, P28, and *E. chaffeensis* DnaK, a chaperone expected to be inaccessible to trypsin (Zhang et al., 2013). Following surface trypsinolysis, immunosignal for P28 as well as the EplA bands, but not DnaK was pronouncedly reduced (Figure 2D). Next, to determine if EplA is expressed and elicits a humoral immune response during natural infection, a panel of sera from client-owned dogs that had previously tested positive for *Ehrlichia* spp. infection or serum from an uninfected dog was screened in an ELISA against His-EplA. All *Ehrlichia* spp.-positive sera recognized recombinant EplA (Figure 2E). These data implicate EplA as a surface-localized protein that *E. chaffeensis* expresses during infection of monocytic host cells in tissue culture and that it or its orthologs are expressed during natural *Ehrlichia* spp. infection in dogs.

### EplA Contributes to *E. chaffeensis* Infection of Host Cells and Interacts With PDI

**Figure 3:**
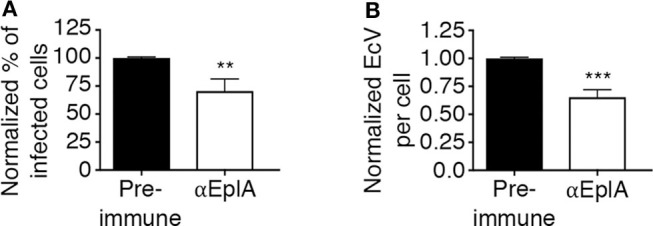
EplA contributes to *E. chaffeensis* infection of host cells. Pretreatment of *E. chaffeensis* with anti-EplA reduces infection of THP-1 cells. Host cell-free *E. chaffeensis* DC organisms were incubated with rat polyclonal antiserum raised against His-EplA or rat preimmune serum. The treated bacteria were incubated with THP-1 cells for 60 min. After removal of unbound bacteria, the infection was allowed to proceed. After 24 h, the cells were fixed and examined by fluorescence microscopy to determine percentages of infected cells **(A)** and numbers of EcVs per cell **(B)**. Data are presented as the mean values ± SD from triplicate samples and are representative of experiments performed three separate times. Statistically significant values are indicated. **P < 0.01; ***P < 0.001.

**Figure 4:**
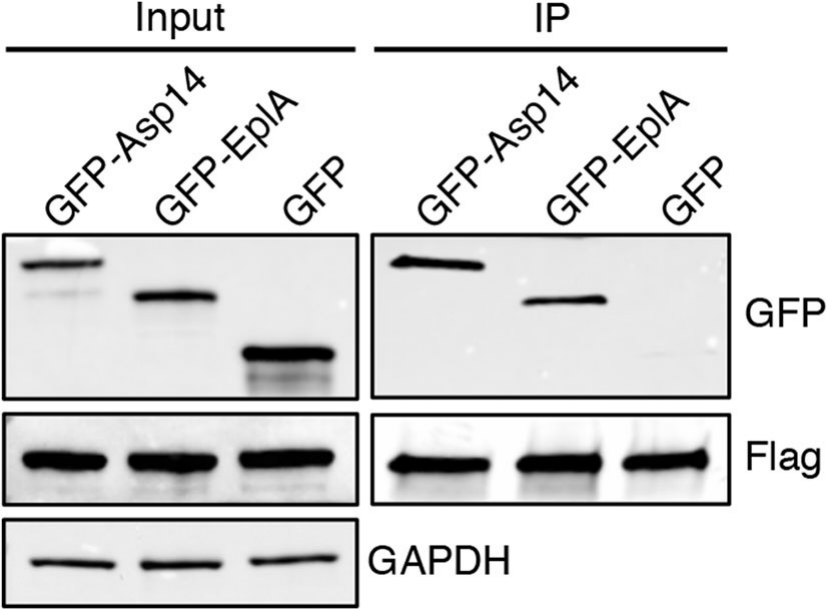
EplA binds PDI. HEK-293T cells were transfected to co-express Flag-PDI and GFP-Asp14, GFP-EplA, or GFP. Input lysates were analyzed by Western blotting with GFP and Flag antibodies to confirm expression of each ectopically expressed protein and with GAPDH antibody to verify that equivalent amounts of protein were in each sample. Flag antibody-conjugated agarose beads were incubated with whole cell lysates to immunoprecipitate (IP) Flag-PDI and its interacting proteins. The resulting Western blots were probed with Flag antibody to confirm that Flag-PDI was recovered and GFP antibody to assess if Flag-PDI co-immunoprecipitated GFP-EplA or GFP. Data are representative of three independent experiments that yielded similar results.

To investigate if EplA participates in *E. chaffeensis* infection of monocytic cells, DC organisms were treated with heat-inactivated EplA antiserum followed by incubation with THP-1 cells, which have been previously verified to have PDI on their surfaces (Langer et al., 2013). At 24 h, the cells were examined by immunofluorescence microscopy for the presence of *E. chaffeensis*-containing vacuoles (EcVs). Anti-EplA reduced the percentage of infected cells and mean number of EcVs by ~30% (Figures 3A,B), thereby confirming that EplA contributes to cellular invasion. Next, the ability of EplA to bind PDI was assessed. Flag-PDI was co-expressed in HEK-293T cells with GFP-EplA, GFP-Asp14 as a positive control, or GFP alone as a negative control. The cells were lysed and incubated with Flag antibody-coated beads to immunoprecipitate Flag-PDI and interacting proteins. Flag-PDI co-immunoprecipitated GFP-EplA and GFP-Asp14, but not GFP (Figure 4). Thus, EplA is capable of interacting with PDI.

### Host Cell Surface PDI and Thioredoxin-1 Thiol Reductase Activity Benefits *E. chaffeensis* Infection

**Figure 5:**
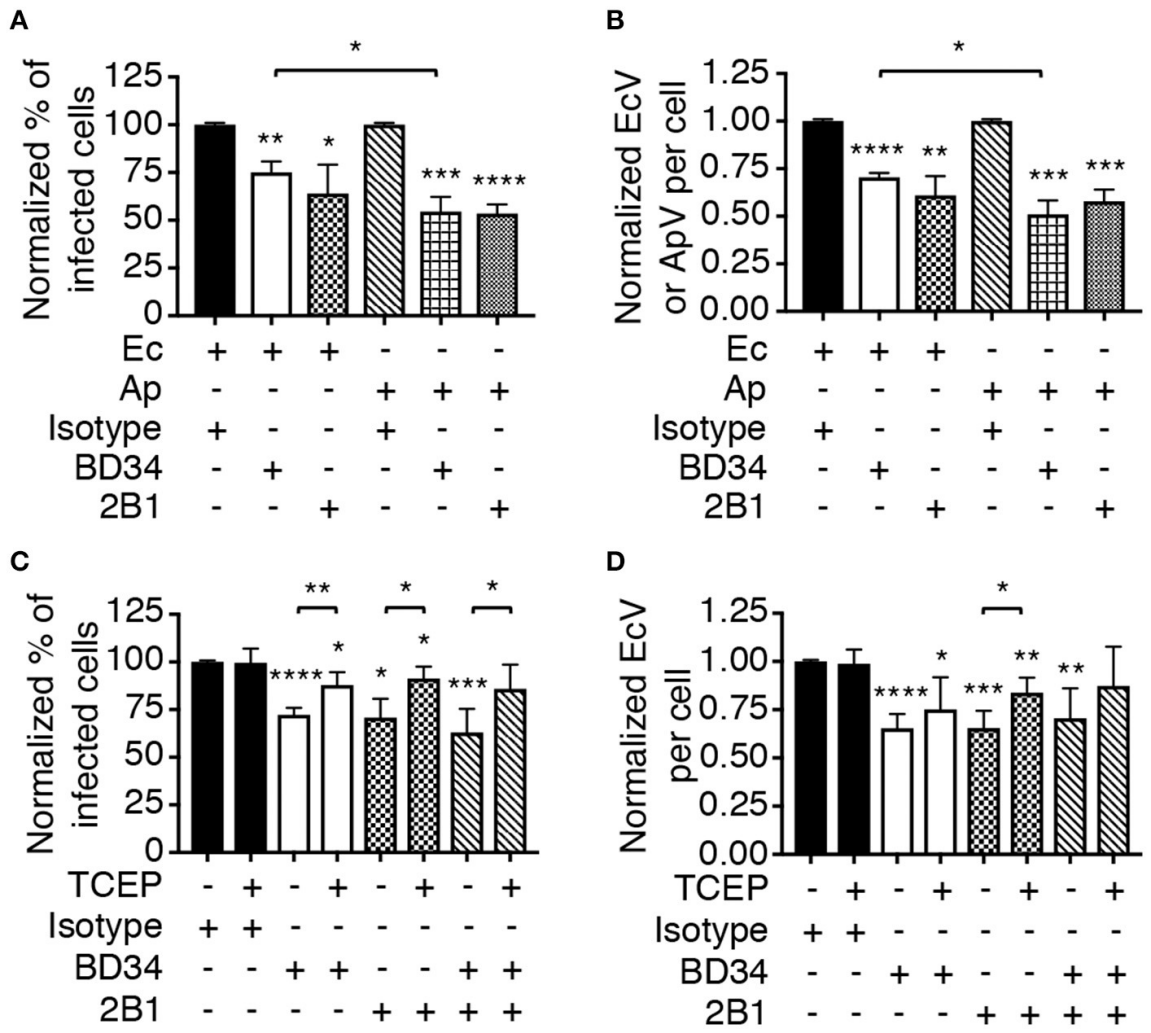
Host cell surface thiol reductase activity benefits *E. chaffeensis* infectivity. **(A,B)** Antibody-mediated inhibition of PDI or Trx1 reductase activity reduces *E. chaffeensis* infection. THP-1 cells were incubated with *E. chaffeensis* organisms in the presence of PDI catalytically neutralizing antibody BD34, Trx1-specific antibody 2B1, or isotype control. As a positive control for the ability of these antibodies to inhibit infection, a parallel experiment was performed in which *A. phagocytophilum* bacteria were incubated with HL-60 cells in the presence of the same antibodies. At 24 h, the cells were examined by immunofluorescence microscopy for the percentages of infected cells **(A)** and numbers of EcVs or ApVs per cell **(B)**. **(C,D)** TCEP nullifies BD34- and 2B1-mediated inhibition of *E. chaffeensis* infection. THP-1 cells that had been treated with BD34, 2B1, both antibodies, or isotype control were incubated with *E. chaffeensis* organisms in the presence or absence of TCEP for 30 min followed by PBS washing. The cells were assessed for the percentage of infected cells **(C)** and number of EcVs per cell **(D)**. All data are shown as the mean ± SD of triplicate samples and are representative of experiments performed a minimum of three times. Statistically significant values are indicated. *P < 0.05; **P < 0.01; ***P < 0.001; ****P < 0.0001. Asterisks above columns indicate statistically significant differences in infection relative to isotype control-treated cells, while asterisks over brackets denote statistically significant difference between the indicated pairs.

Asp14 binding to PDI exploits its thiol reductase activity to facilitate *A. phagocytophilum* cellular invasion (Green et al., 2020). Thioredoxin-1 (Trx1) is another cell surface reductase that contributes to *A. phagocytophilum* infection. It was therefore examined if PDI and Trx1 enzymatic activities are important for *E. chaffeensis* infection. THP-1 cells were treated with monoclonal antibody BD34, which catalytically neutralizes PDI (Popescu et al., 2010), or 2B1, a Trx1-specific antibody that inhibits Trx1 activity-dependent HIV cellular entry (Stantchev et al., 2012). Both antibodies also antagonize *A. phagocytophilum* infection (Green et al., 2020). The THP-1 cells were incubated with *E. chaffeensis* DC bacteria and examined for EcVs 24 h later. As a positive control for the inhibition of infection, BD34-, or 2B1-treated HL-60 cells were incubated with *A. phagocytophilum* and assessed in parallel. Relative to isotype control, BD34 and 2B1 significantly inhibited infection by both pathogens (Figures 5A,B). To further confirm that disulfide bond reduction benefits *E. chaffeensis* infection, THP-1 cells were treated with BD34, 2B1, or isotype control and incubated with DC organisms in the presence of tris(2-carboxyethyl)phosphine-HCl (TCEP) or vehicle. TCEP is a membrane-impermeable reducing agent that reduces disulfide bonds on bacterial and host cells to complement antibody-mediated inactivation of host cell surface thiol reductases (Uehara et al., 2006; Abromaitis and Stephens, 2009; Green et al., 2020). TCEP restored the ability of *E. chaffeensis* to infect THP-1 cells that had been treated with BD34, 2B1, or both antibodies (Figures 5C,D). Thus, PDI and Trx1 reduction of disulfide bonds on the bacterial and/or host cell surface contributes to *E. chaffeensis* infection.

### PDI-Mediated Thiol Reduction at the Host Cell Surface, but Not the Bacterial Surface, Contributes to *E. chaffeensis* Infection

**Figure 6:**
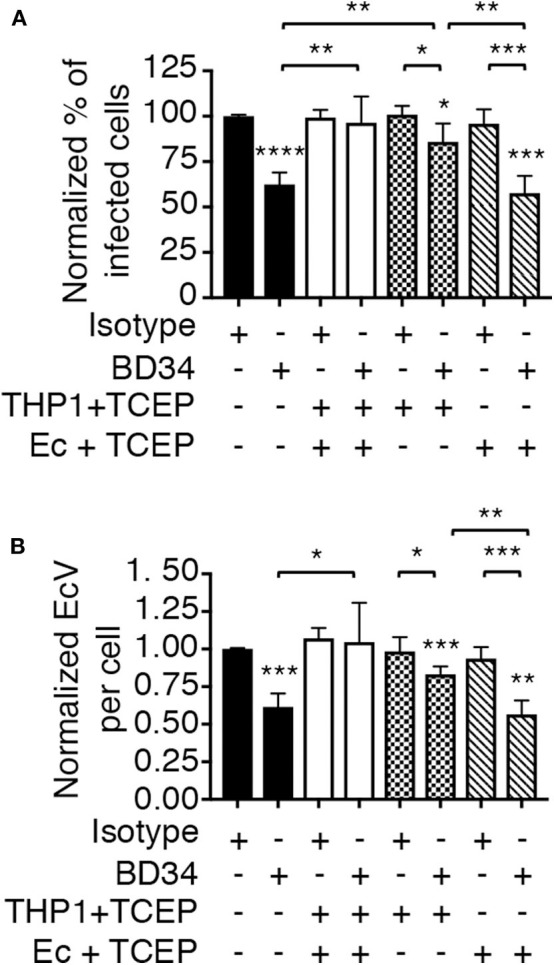
PDI-mediated thiol reduction at the host cell surface contributes to *E. chaffeensis* infection. THP-1 cells were treated with antibody BD34 to inhibit cell surface PDI activity or with isotype control antibody. The THP-1 cells were then incubated in culture medium containing TCEP for 30 min to reduce host cell surface disulfide bonds or culture medium alone followed by incubation with *E. chaffeensis* DC bacteria. Alternatively, DC organisms were treated with TCEP for 30 min to reduce ehrlichial surface disulfide bonds followed by PBS washing and then incubation with BD34- or isotype control-treated THP-1 cells. At 24 h, the host cells were examined using immunofluorescence microscopy to determine the percentage of infected cells **(A)** and number of EcVs per cell **(B)**. Data are the mean ± SD of triplicate samples and are representative of four separate experiments. Statistically significant values are indicated. *P < 0.05; **P < 0.01; ***P < 0.001; ****P < 0.0001. Asterisks above columns indicate statistically significant differences in infection relative to isotype control-treated cells, while asterisks over brackets denote statistically significant difference between the indicated pairs.

To determine if PDI-mediated thiol reduction of a host or bacterial surface protein benefits *E. chaffeensis* infection, the TCEP restoration of infectivity assay was repeated with two additional conditions. First, BD34-treated THP-1 cells were exposed to TCEP to reduce disulfide bonds exclusively on host cell surfaces followed by PBS washing to remove TCEP and then incubation with untreated *E. chaffeensis* DCs. Second, DCs were exposed to TCEP, washed, and subsequently incubated with BD34-treated THP-1 cells so that only ehrlichial surface disulfide bonds were reduced. TCEP treatment of THP-1 cells alone, but not *E. chaffeensis* alone rescued the bacterium's ability to infect BD34-treated host cells (Figure 6). These results demonstrate that PDI-catalyzed disulfide bond reduction of one or more host cell surface proteins is important for *E. chaffeensis* infection.

### Antibody Specific for the EplA C-Terminus Impairs the Ability of *E. chaffeensis* to co-opt PDI Thiol Reductase Activity

**Figure 7:**
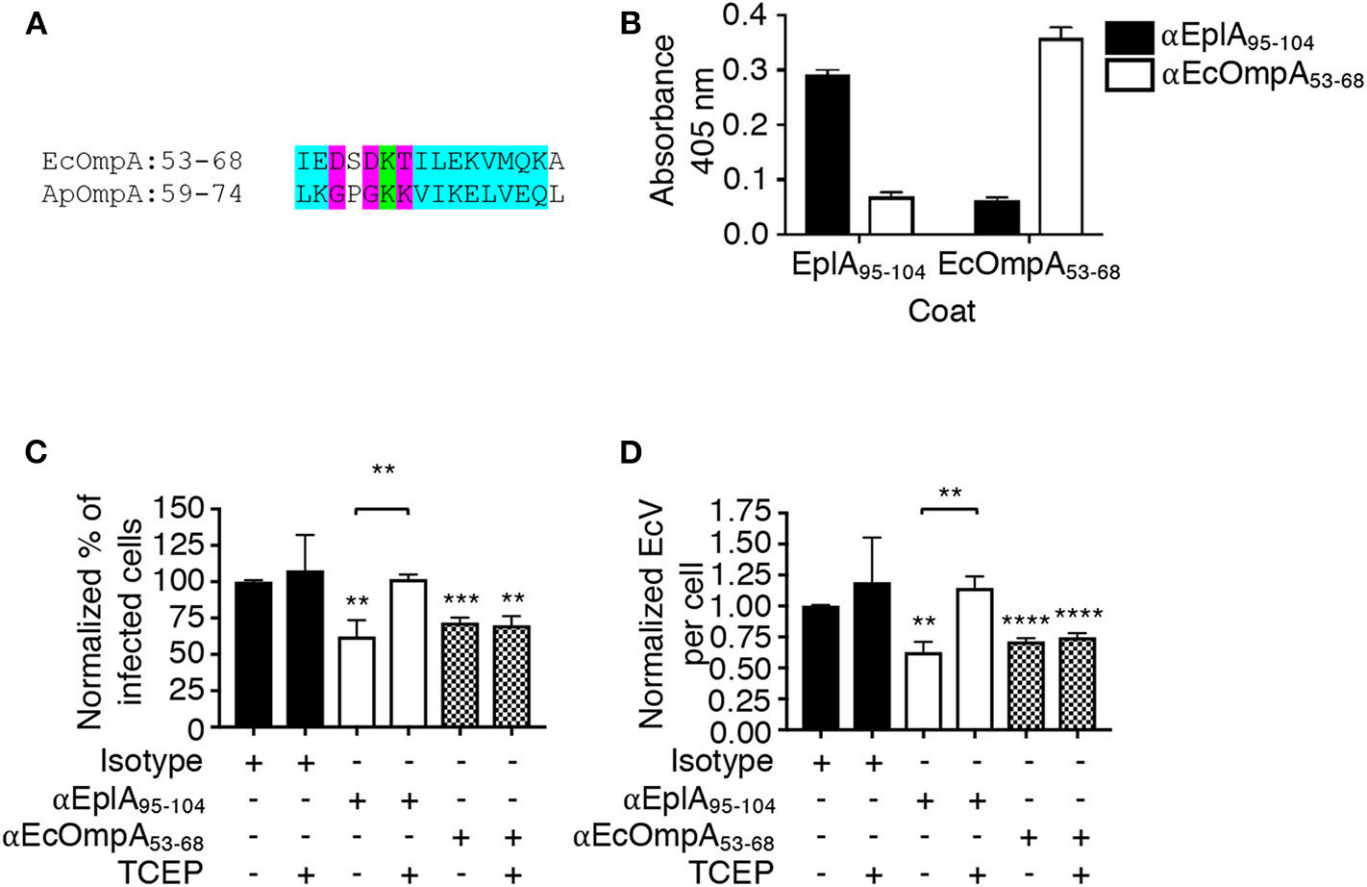
EplA residues 95-104 are key for *E. chaffeensis* to co-opt host cell surface PDI thiol reductase activity. **(A)** Alignment of the ApOmpA_59−74_ receptor binding domain with EcOmpA_53−68_. Green highlighting denotes identical amino acids. Turquoise highlighting demarcates highly similar amino acids as defined by Clustal Omega. Magenta highlighting signifies amino acids that are weakly similar. **(B)** ELISA in which EplA_95−104_ and EcOmpA_53−68_ antibodies were used to screen wells coated with peptides corresponding to EplA_95−104_ and EcOmpA_53−68_. Each antiserum only recognized the peptide against which it had been raised. **(C,D)** *E. chaffeensis* DC organisms were treated with rabbit preimmune serum or rabbit antiserum (α) specific for EplA_95−104_ or EcOmpA_53−68_ followed by incubation with THP-1 cells in the continued presence of antiserum with or without TCEP. The host cells were examined 24 h later by fluorescence microscopy for the percentages of infected cells **(C)** and numbers of EcVs per cell **(D)**. Data are presented as the mean values ± SD from triplicate samples and are representative of experiments performed three separate times. Statistically significant values are indicated. **P < 0.01; ***P < 0.001; ****P < 0.0001. Asterisks above columns indicate statistically significant differences in levels of infection relative to the results for preimmune serum-treated cells. Asterisks over the brackets denote a statistically significant difference between the levels of infection of EplA_95−104_ antiserum-treated DCs following incubation with or without TCEP.

The ability of *A. phagocytophilum* Asp14 to engage PDI and co-opt its thiol reductase activity is dependent on its C-terminal domain, which is encompassed by residues 113-124 and exhibits homology to the EplA residues 95-104 (EplA_95−104_) (Figure 1). Accordingly, it was next examined if EplA_95−104_ contributes to *E. chaffeensis* infection and if it is important for co-opting host cell surface thiol reductase activity. DC organisms were treated with preimmune serum or antiserum specific for EplA_95−104_ or *E. chaffeensis* OmpA residues 53-68 (EcOmpA_53−68_) followed by incubation with THP-1 cells in the presence or absence of TCEP. Anti-EcOmpA_53−68_ was included as a control because EcOmpA participates in *E. chaffeensis* infection and is homologous to the *A. phagocytophilum* adhesin, OmpA (ApOmpA) (Cheng et al., 2011; Ojogun et al., 2012). The EcOmpA functional domain has not yet been defined, but its residues 53-68 are homologous to the ApOmpA_59−74_ receptor binding domain (Ojogun et al., 2012; Seidman et al., 2015) (Figure 7A). The specificity of EplA_95−104_ and EcOmpA_63−58_ antisera for their respective antigens was confirmed by ELISA (Figure 7B). Each antiserum significantly reduced *E. chaffeensis* infection (Figures 7C,D). However, TCEP restored *E. chaffeensis* infectivity in the presence of anti-EplA_95−104_, but not anti-EcOmpA_53−68_. Thus, EplA_95−104_ and EcOmpA_53−68_ each contribute to *E. chaffeensis* infection, but only EplA_95−104_ does so by exploiting host cell surface PDI-mediated disulfide reduction. Additionally, because TCEP did not nullify the ability of EcOmpA_53−68_ antiserum to inhibit ehrlichial infection, it can be inferred that the TCEP-mediated rescue of infectivity in the presence of anti-EplA_95−104_ as well as BD34 and 2B1 was not simply due to antibody inactivation.

## Discussion

This study demonstrates that EplA binds PDI on monocytic cell surfaces to exploit the enzyme's thiol reductase activity to mediate *E. chaffeensis* cellular invasion. The mechanism is thematically similar to that orchestrated by the EplA *A. phagocytophilum* ortholog, Asp14 (Green et al., 2020). Both adhesins are expressed during natural infection and their PDI binding domains are C-terminally located. However, whereas Asp14 binding to PDI brings it and the *A. phagocytophilum* surface in sufficient proximity to mediate reduction of bacterial OMP disulfide bonds as a critical step in infection (Green et al., 2020), EplA engages PDI to facilitate reduction of host cell surface disulfide bonds to promote infection. Thus, while the two *Anaplasmataceae* adhesins have evolved to co-opt host cell PDI enzymatic activity, they do so by distinct mechanisms. The host and *A. phagocytophilum* proteins that are reduced during EplA- and Asp14-induced invasion, respectively, remain unidentified. The observed reduction in EplA levels following trypsinolysis of *E. chaffeensis* DC organisms confirms that EplA is present on the bacterial surface as has been shown for Asp14 (Kahlon et al., 2013). Also, like Asp14, EplA is not predicted to carry a canonical signal sequence or a transmembrane domain (Kahlon et al., 2013). How either adhesin is translocated to or associates with the bacterial outer membrane is unclear. Neither exhibits homology to known crystal structures. EplA and Asp14 might be anchored to the cell wall via a posttranslational modification or could simply be peripheral membrane proteins.

Antibody-mediated neutralization of PDI or Trx1 each inhibits *E. chaffeensis* infection comparably and in a TCEP-reversible manner. Thus, the EplA-PDI interaction could bring the host cell substrate close enough to PDI or Trx1 such that it becomes reduced. Alternatively, EplA could interact with a domain that is shared between the two enzymes. If so, this is presumably a conformational domain because, while members of the thioredoxin superfamily do not have a high level of sequence similarity, they are structurally similar (Carvalho et al., 2006). The reduction in the *E. chaffeensis* load achieved by anti-EplA_95−104_, BD34, 2B1, or both BD34 and 2B1 does not exceed more than ~40%, which supports that this pathogen's cellular invasion is multifactorial and involves redundant/complementary entry routes. Indeed, at least three other *E. chaffeensis* proteins—EtpE (entry triggering protein of *Ehrlichia*), TRP120 (120-kDa tandem-repeat protein), and EcOmpA—also facilitate infection (Popov et al., 2000; Cheng et al., 2011; Mohan Kumar et al., 2013, 2015; Luo et al., 2015). Like EplA, they are expressed by the infectious DC form (Popov et al., 2000; Cheng et al., 2011; Mohan Kumar et al., 2013). Their expression is regulated by the two-component response regulator, CtrA (Cheng et al., 2011; Mohan Kumar et al., 2013). Whether CtrA controls EplA expression is unknown. EtpE engages glycophosphotidylinositol-anchored DNAse X, CD147, and heterogenous nuclear ribonuclear protein K (hnRNP-K) (Mohan Kumar et al., 2013). The EtpE-hnRNP-K interaction activates neuronal Wiskott-Aldrich syndrome protein to mobilize actin polymerization-dependent *E. chaffeensis* internalization (Mohan Kumar et al., 2015). TRP120 is a multifunctional effector that mediates uptake by activating canonical/β-catenin-dependent and non-canonical/β-catenin-independent Wnt pathways (Luo et al., 2015). EcOmpA is involved in ehrlichial cellular adherence and invasion, but its cognate receptor is undefined (Cheng et al., 2011). The identified EtpE and TRP120 receptors are not known to be modified by cell surface PDI. However, PDI, hnRNP-K, and E-selectin, which is a putative *E. chaffeensis* receptor, all localize to lipid rafts in monocytes (Zhang et al., 2003, 2008). *E. chaffeensis* entry into monocytes depends on lipid rafts and GPI-anchored proteins like DNAse X (Lin and Rikihisa, 2003; Mohan Kumar et al., 2015). Given that dengue virus utilizes PDI to enter monocytes via lipid rafts (Diwaker et al., 2015), our data and these reports collectively implicate the EplA receptor, PDI, as part of a complex of *E. chaffeensis* receptors that cluster in lipid rafts.

This study identifies EplA_95−104_ as being important for binding PDI and EcOmpA_53−68_ as being critical for EcOmpA-mediated infection. EcOmpA_53−68_ is homologous to *A. phagocytophilum* ApOmpA_59−74_ and *A. marginale* OmpA_53−68_, which bind sialyl Lewis x and structurally similar sialylated and fucosylated glycans, respectively (Seidman et al., 2015; Hebert et al., 2017). Considering the similarity among these three domains, perhaps EcOmpA also binds to a similar glycan on monocytes. TCEP nullified the inhibitory effect on *E. chaffeensis* infectivity achieved by anti-EplA_95−104_, but not anti-EcOmpA_53−68_ thereby confirming that the ability to co-opt PDI activity is EplA-specific. Nonetheless, these data identify two linear peptide determinants that can be targeted to inhibit *E. chaffeensis* infection. While the data presented herein collectively argue that EplA or EplA_95−104_ antibodies inhibit *E. chaffeensis* infection by specifically antagonizing the EplA-PDI interaction, it cannot be ruled out that inhibition could also occur via the antibodies bound to the bacterial surface sterically hindering pathogen-host cell interactions or by promoting Fcγ receptor-mediated phagocytosis by THP-1 cells.

In closing, this report identifies EplA as a novel adhesin that exploits cell surface PDI to promote *E. chaffeensis* infection of monocytes, demonstrates that this general strategy is shared between this pathogen and *A. phagocytophilum* but the two mechanisms are distinct from each other, delineates the EplA and EcOmpA functional domains, and establishes them as potential protective antigens.

## Materials and Methods

### Cell Lines and Cultivation of *E. chaffeensis* and *A. phagocytophilum*

*E. chaffeensis* str. Arkansas was kindly provided by Dr. J. Stephen Dumler (Uniformed Services University of the Health Sciences). Uninfected non-adherent human monocytic THP-1 cells [TIB-202; American Type Culture Collection (ATCC), Manassas, VA] were cultivated in RPMI 1640 medium containing 1% L-glutamine (Invitrogen, Carlsbad, CA) and supplemented with 10% fetal bovine serum (FBS) (Gemini Bio-Products, West Sacramento, CA) at 37°C in a humified incubator in 5% atmospheric CO_2_. *E. chaffeensis*-infected THP-1 cells were grown in RPMI 1640 medium containing 1% L-glutamine and 5% FBS. Uninfected and *A. phagocytophilum* str. NCH-1-infected HL-60 cells (CCL-240, ATCC) and human embryonic kidney HEK-293T cells were cultured as described previously (Truchan et al., 2016; Green et al., 2020).

### Commercial Antibodies, Chemicals, and Reagents

Commercial antibodies were BD34 (product number 610947; BD Biosciences, San Jose, CA); 2B1 (MA1-82452; ThermoFisher Scientific, Rockford, IL); mouse IgG1 isotype control (554121; BD Biosciences); anti-GFP (product number A6455; Invitrogen); anti-Flag (F7425; Invitrogen); Alexa Fluor 488-conjugated goat anti-rat IgG and goat anti-rabbit IgG (A21210 and A11034, respectively; Invitrogen); Alexa Fluor 594-conjugated goat anti-rat IgG (A21471, Invitrogen); horseradish peroxidase-conjugated goat anti-mouse IgG, anti-rabbit IgG, and anti-rat IgG (7076, 7074, and 7077, respectively; Cell Signaling Technology, Danvers, MA); and anti-6X-His tag (372900, Invitrogen) M2 Flag affinity resin was purchased from MilliporeSigma (Burlington, MA). TCEP, Lipofectamine 2000, and protein A/G agarose resin were obtained from ThermoFisher Scientific.

### Plasmids, Recombinant Protein Expression, Antisera Generation, and Western Blot Analysis

GFP-Asp14 and Flag-PDI expressing plasmids were produced previously (Green et al., 2020). A pUC57 vector carrying mammalian codon-optimized DNA sequences encoding full-length EplA (UniProtKB identification number Q2GH86) with flanking 5′ EcoRI and 3′ SalI restriction sites was provided by Genscript (Piscataway, NJ). The insert sequence was excised by restriction enzyme digestion using EcoRI and SalI and ligated into pEGFP-C1 (Clontech, Palo Alto, CA) as described previously (Viebrock et al., 2014). The EplA coding sequence was provided by Genscript in pET45b(+) (MilliporeSigma). The constructs encoding His-tagged EplA and P28 were transformed into *E. coli* BL21 (DE3) for protein production, and purification by immobilized metal-affinity chromatography as described (Mcdowell et al., 2009; Miller et al., 2011). His-EplA and His-P28 were used for generating rat polyclonal antisera as described previously (Izac et al., 2020) and was performed under the approval of the Institutional Animal Care and Use Committee at Virginia Commonwealth University (protocol number AM10000387). Keyhole limpet hemocyanin-conjugated peptides corresponding to EplA_95−104_ and ECH_0462_53−68_ were synthesized and injected into rabbits to yield monospecific antiserum against each antigen by New England Peptide (Gardner, MA). SDS-PAGE and Western blot analyses were performed as previously described (Viebrock et al., 2014; Evans et al., 2018). Immunoreactive band size was determined using Image Lab 6.0 software (BioRad).

### Preparation of *E. chaffeensis* DC and RC Organisms and Surface Trypsinolysis

Heavily infected (>80%) THP-1 cells and any ehrlichiae in the culture medium were pelleted by centrifugation at 5,200 g for 15 min at 4°C. The pellet was resuspended in 6 ml of ice-cold PBS. To isolate host cell-free *E. chaffeensis* DC and RC bacteria, the suspension was repeatedly passed through a 27-guage blunt-ended needle. Bacteria were separated from unbroken host cells and debris by centrifugation at 750 g for 5 min at 4°C. The supernatant was subjected to two rounds of centrifugation at 1,000 g for 6 min at 4°C, each time transferring the supernatant to a new tube. The resulting supernatant was transferred to a new tube and subjected to a centrifugation at 5,200 g for 6 min at 4°C. To isolate DC organisms, the infected THP-1 cell suspension in PBS was subjected to four 8-s bursts on ice interspersed with 8-s rest periods using a Misonix S4000 ultrasonic processor (Farmingdale, NY) on an amplitude setting of 30, which destroys host cells and RCs but not DCs (Huang et al., 2010). The resulting *E. chaffeensis* pellet was washed three times with ice-cold PBS and either resuspended in sample buffer for Western blot analyses or resuspended in PBS and used for cellular infection assays. *E. chaffeensis* DC organisms were subjected to surface trypsinolysis as described previously for *A. phagocytophilum* DC bacteria (Ojogun et al., 2012).

### *E. chaffeensis* and *A. phagocytophilum* Cellular Infection Assays

*E. chaffeensis* DC organisms that had been recovered from 1 × 10^6^ infected THP-1 cells were incubated with 250,000 naïve THP-1 cells in 100 μl of culture medium for 1 h at 37°C in a humidified incubator with 5% atmospheric CO_2_ with gentle agitation every 10 min. Unbound DCs were removed by washing three times with PBS using 300 g centrifugation steps at room temperature and discarding the supernatant. The cells were resuspended in RPMI 1640 containing 10% (vol/vol) FBS and incubated at 37°C in a humidified incubator with 5% atmospheric CO_2_. At 24 h post-infection, the cells were washed, screened with DAPI to label intracellular *E. chaffeensis* bacteria, and examined by immunofluorescence microscopy to determine the percentage of infected cells and number of EcVs per cell as previously described (Ojogun et al., 2012). To determine the relevance of EplA, EplA_95−104_, and EcOmpA_53−68_ to infection, *E. chaffeensis* DCs were incubated with antisera specific for these antigens or preimmune serum for 1 h on ice prior to being added to THP-1 cells. To determine the contribution of host cell surface disulfide reductase activity to infection, THP-1 cells were treated with 10 μg ml^−1^ BD34, 2B1, both antibodies, or isotype control in RPMI 1640 containing 10% (vol/vol) FBS for 1 h at 37°C, followed by incubation with *E. chaffeensis* DC bacteria. In some cases, antibody-treated THP-1 cells were incubated with ehrlichial DCs followed by the addition of TCEP in culture medium to a final concentration of 0.01 mM for 30 min at 37°C prior to PBS washing and processing for immunofluorescence microscopy. In other instances, either antibody-treated THP-1 cells or untreated DCs were incubated in culture medium containing 0.01 mM TCEP at 37°C for 30 min followed by PBS washing and subsequent incubation with each other. Some experiments were performed in parallel in which BD34, 2B1, or isotype control were used to treat HL-60 cells followed by incubation with *A. phagocytophilum* DC organism and subsequent assessment for infection as previously described (Ojogun et al., 2012; Green et al., 2020).

### Immunoprecipitation

HEK-293T cells grown in six-well-plates to 80% confluency were co-transfected with 2 μg each of plasmid encoding GFP, GFP-EplA, or GFP-Asp14 together with 2 μg plasmid encoding Flag-PDI using Lipofectamine 2000 as described (Evans et al., 2018). Sixteen hours post-transfection, the cells were lysed and Flag-PDI and interacting proteins were precipitated using Flag-affinity agarose resin (MilliporeSigma) as described (Beyer et al., 2017). Eluates were resolved by SDS-PAGE in 4–20% mini-Protean gels (Bio-Rad, Hercules, CA) as described (Viebrock et al., 2014). Western blot analyses were performed as described (Evans et al., 2018) using GFP and Flag tag primary antibodies at a 1:1,000 dilution and HRP-conjugated secondary antibodies at a 1:10,000 dilution. Input lysates were subjected to Western blot analysis using GAPDH antibody at a 1:2,500 dilution to confirm that immunoprecipitations were performed using equivalent amounts of lysate per sample.

### ELISA

Polystyrene 96-well-microplates (Corning, Corning, NY) were coated with 500 ng of His-EplA per well in bicarbonate buffer (15 mM sodium carbonate, 24.9 mM sodium bicarbonate; pH 9.6) overnight at 4°C followed by blocking with 5% (vol/vol) non-fat dry milk in PBS with 0.5% Tween-20 (PBST) for 2 h at room temperature. The plates were then incubated with canine sera that had tested positive for *Ehrlichia* spp. infection or serum from an uninfected dog. In some cases, the plates were coated with 500 ng EplA_95−104_ or EcOmpA_53−68_ peptide and screened with rabbit antiserum that had been raised against either peptide conjugated to KLH or preimmune serum. Serum samples were diluted 1:100 in 5% (vol/vol) non-fat dry milk in PBST for 1 h at room temperature, washed three times with PBST, incubated with HRP-conjugated anti-mouse or anti-rabbit IgG at a 1:15,000 dilution at room temperature, and washed again. ABTS (2, 2'-Azino-bis (3-ethylbenzothiazoline-6-sulfonic acid) diammonium salt) substrate (Sigma-Aldrich, St. Louis, MO) was added in the presence of citrate buffer (10.4 mM citric acid, pH 4.1) and 30% (vol/vol) hydrogen peroxide (Sigma-Aldrich) for 30 min followed by absorbance reading at 405 nm in an ELx 808 plate reader (Fisher Scientific, Hanover Park, IL).

### Sequence Alignment

EplA, Ecaj_0636, and Asp14 were aligned using Clustal Omega (https://www.ebi.ac.uk/Tools/msa/clustalo/). The resulting alignment was manually adjusted to align Asp14 residues 115-124, Ecaj_0636 residues 91-98, and EplA residues 95-104 based on the conservation of functionally essential Asp14 residues 121-123 (Kahlon et al., 2013; Green et al., 2020) between the proteins.

### Statistical Analyses

Statistical analyses were performed using the Prism 7.0 software package (GraphPad, San Diego, CA). The student's *t*-test was used to test for a significant difference among pairs. Statistical significance was set at *P* < 0.05.

## Data Availability Statement

The raw data supporting the conclusions of this article will be made available by the authors, without undue reservation.

## Ethics Statement

The animal study was reviewed and approved by Virginia Commonwealth University Institutional Animal Care and Use Committee.

## Author Contributions

JC, RG, and RM conceived and coordinated the studies and wrote the manuscript. RG performed most of the experiments. JI and NO'B cloned, expressed, and purified recombinant EplA and used it to immunize rats for the purpose of generating EplA antiserum. WN performed ELISA, Western blot, and immunofluroescence assays. EB isolated serum from client-owned dogs. All authors contributed to the article and approved the submitted version.

## Conflict of Interest

The authors declare that the research was conducted in the absence of any commercial or financial relationships that could be construed as a potential conflict of interest.

## References

[B1] Abromaitis S. Stephens R. S. (2009). Attachment and entry of Chlamydia have distinct requirements for host protein disulfide isomerase. PLoS Pathog. 5:e1000357. 10.1371/journal.ppat.1000357 19343202 PMC2655716

[B2] Ali Khan H. Mutus B. (2014). Protein disulfide isomerase a multifunctional protein with multiple physiological roles. Front. Chem. 2:70. 10.3389/fchem.2014.00070 25207270 PMC4144422

[B3] Anderson B. E. Dawson J. E. Jones D. C. Wilson K. H. (1991). Ehrlichia chaffeensis, a new species associated with human ehrlichiosis. J. Clin. Microbiol. 29, 2838–2842. 10.1128/JCM.29.12.2838-2842.1991 1757557 PMC270443

[B4] Barbouche R. Miquelis R. Jones I. M. Fenouillet E. (2003). Protein-disulfide isomerase-mediated reduction of two disulfide bonds of HIV envelope glycoprotein 120 occurs Post-CXCR4 binding and is required for fusion. J. Biol. Chem. 278, 3131–3136. 10.1074/jbc.M205467200 12218052

[B5] Beyer A. R. Rodino K. G. Viebrock L. Green R. S. Tegels B. K. Oliver L. D.. (2017). Orientia tsutsugamushi Ank9 is a multifunctional effector that utilizes a novel GRIP-like Golgi localization domain for Golgi-to-endoplasmic reticulum trafficking and interacts with host COPB2. Cell. Microbiol. 19:e12727. 10.1111/cmi.12727 28103630 PMC5469707

[B6] Carvalho A. P. Fernandes P. A. Ramos M. J. (2006). Similarities and differences in the thioredoxin superfamily. Prog. Biophys. Mol. Biol. 91, 229–248. 10.1016/j.pbiomolbio.2005.06.012 16098567

[B7] Centers for Disease Control Prevention (2020). Ehrlichiosis: Epidemiology and Statistics. Available online at: www.cdc.gov/ehrlichiosis/stats (accessed May 28, 2020).

[B8] Cheng Z. Miura K. Popov V. L. Kumagai Y. Rikihisa Y. (2011). Insights into the CtrA regulon in development of stress resistance in obligatory intracellular pathogen Ehrlichia chaffeensis. Mol. Microbiol. 82, 1217–1234. 10.1111/j.1365-2958.2011.07885.x 22014113 PMC3241975

[B9] Diwaker D. Mishra K. P. Ganju L. Singh S. B. (2015). Protein disulfide isomerase mediates dengue virus entry in association with lipid rafts. Viral Immunol. 28, 153–160. 10.1089/vim.2014.0095 25664880

[B10] Evans S. M. Rodino K. G. Adcox H. E. Carlyon J. A. (2018). Orientia tsutsugamushi uses two Ank effectors to modulate NF-kappaB p65 nuclear transport and inhibit NF-kappaB transcriptional activation. PLoS Pathog. 14:e1007023. 10.1371/journal.ppat.1007023 29734393 PMC5957444

[B11] Fischer A. Rudel T. (2018). Safe haven under constant attack-the Chlamydia-containing vacuole. Cell. Microbiol. 20:e12940. 10.1111/cmi.12940 30101516

[B12] Gallina A. Hanley T. M. Mandel R. Trahey M. Broder C. C. Viglianti G. A.. (2002). Inhibitors of protein-disulfide isomerase prevent cleavage of disulfide bonds in receptor-bound glycoprotein 120 and prevent HIV-1 entry. J. Biol. Chem. 277, 50579–50588. 10.1074/jbc.M204547200 12218051

[B13] Green R. S. Naimi W. A. Oliver L. D. Jr. O'bier N. Cho J. Conrad D. H.. (2020). Binding of host cell surface protein disulfide isomerase by *Anaplasma phagocytophilum* Asp14 enables pathogen infection. MBio 11:e03141–19. 10.1128/mBio.03141-19 31992623 PMC6989111

[B14] Hebert K. S. Seidman D. Oki A. T. Izac J. Emani S. Oliver L. D. Jr.. (2017). Anaplasma marginale outer membrane protein a is an adhesin that recognizes sialylated and fucosylated glycans and functionally depends on an essential binding domain. Infect. Immun. 85:e00968-16. 10.1128/IAI.00968-16 27993973 PMC5328490

[B15] Heinzen R. A. Hackstadt T. Samuel J. E. (1999). Developmental biology of Coxiella burnettii. Trends Microbiol. 7, 149–154. 10.1016/S0966-842X(99)01475-4 10217829

[B16] Hidalgo M. Vesga J. F. Lizarazo D. Valbuena G. (2009). A survey of antibodies against Rickettsia rickettsii and Ehrlichia chafeensis in domestic animals from a rural area of Colombia. Am. J. Trop. Med. Hyg. 80, 1029–1030. 10.4269/ajtmh.2009.80.1029 19478270

[B17] Hotopp J. C. Lin M. Madupu R. Crabtree J. Angiuoli S. V. Eisen J.. (2006). Comparative genomics of emerging human ehrlichiosis agents. PLoS Genet. 2:e21. 10.1371/journal.pgen.0020021 16482227 PMC1366493

[B18] Huang B. Troese M. J. Howe D. Ye S. Sims J. T. Heinzen R. A.. (2010). *Anaplasma phagocytophilum* APH_0032 is expressed late during infection and localizes to the pathogen-occupied vacuolar membrane. Microb. Pathog. 49, 273–284. 10.1016/j.micpath.2010.06.009 20600793 PMC2919654

[B19] Ismail N. Mcbride J. W. (2017). Tick-borne emerging infections: ehrlichiosis and anaplasmosis. Clin. Lab. Med. 37, 317–340. 10.1016/j.cll.2017.01.006 28457353

[B20] Izac J. R. O'bier N. S. Oliver L. D. Jr. Camire A. C. Earnhart C. G. Leblanc Rhodes D. V.. (2020). Development and optimization of OspC chimeritope vaccinogens for Lyme disease. Vaccine 38, 1915–1924. 10.1016/j.vaccine.2020.01.027 31959423 PMC7085410

[B21] Jiang X. M. Fitzgerald M. Grant C. M. Hogg P. J. (1999). Redox control of exofacial protein thiols/disulfides by protein disulfide isomerase. J. Biol. Chem. 274, 2416–2423. 10.1074/jbc.274.4.2416 9891011

[B22] Kahlon A. Ojogun N. Ragland S. A. Seidman D. Troese M. J. Ottens A. K.. (2013). *Anaplasma phagocytophilum* Asp14 is an invasin that interacts with mammalian host cells via its C terminus to facilitate infection. Infect. Immun. 81, 65–79. 10.1128/IAI.00932-12 23071137 PMC3536139

[B23] Kocan K. M. Yellin T. N. Claypool P. L. Barron S. J. Ewing S. A. Hair J. A. (1990). Development and infectivity of Anaplasma marginale in *Dermacentor andersoni* nymphs. Am. J. Vet. Res. 51, 1292–1294. 2386330

[B24] Kocan K. M. Yellin T. N. Ewing S. A. Hair J. A. Barron S. J. (1984). Morphology of colonies of Anaplasma marginale in nymphal *Dermacentor andersoni*. Am. J. Vet. Res. 45, 1434–1440. 24049913

[B25] Langer F. Spath B. Fischer C. Stolz M. Ayuk F. A. Kroger N.. (2013). Rapid activation of monocyte tissue factor by antithymocyte globulin is dependent on complement and protein disulfide isomerase. Blood 121, 2324–2335. 10.1182/blood-2012-10-460493 23315166 PMC3606067

[B26] Lin M. Rikihisa Y. (2003). Obligatory intracellular parasitism by *Ehrlichia chaffeensis* and *Anaplasma phagocytophilum* involves caveolae and glycosylphosphatidylinositol-anchored proteins. Cell. Microbiol. 5, 809–820. 10.1046/j.1462-5822.2003.00322.x 14531896

[B27] Luo T. Dunphy P. S. Lina T. T. Mcbride J. W. (2015). *Ehrlichia chaffeensis* exploits canonical and noncanonical host wnt signaling pathways to stimulate phagocytosis and promote intracellular survival. Infect. Immun. 84, 686–700. 10.1128/IAI.01289-15 26712203 PMC4771358

[B28] Maeda K. Markowitz N. Hawley R. C. Ristic M. Cox D. Mcdade J. E. (1987). Human infection with *Ehrlichia canis*, a leukocytic rickettsia. N. Engl. J. Med. 316, 853–856. 10.1056/NEJM198704023161406 3029590

[B29] Mcdowell J. V. Huang B. Fenno J. C. Marconi R. T. (2009). Analysis of a unique interaction between the complement regulatory protein factor H and the periodontal pathogen *Treponema denticola*. Infect. Immun. 77, 1417–1425. 10.1128/IAI.01544-08 19204088 PMC2663137

[B30] Miller D. P. Mcdowell J. V. Bell J. K. Marconi R. T. (2011). Crystallization of the factor H-binding protein, FhbB, from the periopathogen *Treponema denticola*. Acta Crystallogr. Sect. F Struct. Biol. Cryst. Commun. 67, 678–681. 10.1107/S1744309111011298 21636910 PMC3107141

[B31] Mohan Kumar D. Lin M. Xiong Q. Webber M. J. Kural C. Rikihisa Y. (2015). EtpE binding to DNase X induces ehrlichial entry via CD147 and hnRNP-K recruitment, followed by mobilization of N-WASP and Actin. MBio 6, e01541–e01515. 10.1128/mBio.01541-15 26530384 PMC4631803

[B32] Mohan Kumar D. Yamaguchi M. Miura K. Lin M. Los M. Coy J. F.. (2013). *Ehrlichia chaffeensis* uses its surface protein EtpE to bind GPI-anchored protein DNase X and trigger entry into mammalian cells. PLoS Pathog. 9, e1003666. 10.1371/journal.ppat.1003666 24098122 PMC3789761

[B33] Ojogun N. Kahlon A. Ragland S. A. Troese M. J. Mastronunzio J. E. Walker N. J.. (2012). *Anaplasma phagocytophilum* outer membrane protein A interacts with sialylated glycoproteins to promote infection of mammalian host cells. Infect. Immun. 80, 3748–3760. 10.1128/IAI.00654-12 22907813 PMC3486060

[B34] Olano J. P. Masters E. Hogrefe W. Walker D. H. (2003). Human monocytotropic ehrlichiosis, Missouri. Emerging Infect. Dis. 9, 1579–1586. 10.3201/eid0912.020733 14720399 PMC3034327

[B35] Ou W. Silver J. (2006). Role of protein disulfide isomerase and other thiol-reactive proteins in HIV-1 envelope protein-mediated fusion. Virology 350, 406–417. 10.1016/j.virol.2006.01.041 16507315

[B36] Paddock C. D. Childs J. E. (2003). *Ehrlichia chaffeensis*: a prototypical emerging pathogen. Clin. Microbiol. Rev. 16, 37–64. 10.1128/CMR.16.1.37-64.2003 12525424 PMC145301

[B37] Popescu N. I. Lupu C. Lupu F. (2010). Extracellular protein disulfide isomerase regulates coagulation on endothelial cells through modulation of phosphatidylserine exposure. Blood 116, 993–1001. 10.1182/blood-2009-10-249607 20448108 PMC2924232

[B38] Popov V. L. Yu X. Walker D. H. (2000). The 120 kDa outer membrane protein of *Ehrlichia chaffeensis*: preferential expression on dense-core cells and gene expression in *Escherichia coli* associated with attachment and entry. Microb. Pathog. 28, 71–80. 10.1006/mpat.1999.0327 10644493

[B39] Reiser K. Francois K. O. Schols D. Bergman T. Jornvall H. Balzarini J.. (2012). Thioredoxin-1 and protein disulfide isomerase catalyze the reduction of similar disulfides in HIV gp120. Int. J. Biochem. Cell Biol. 44, 556–562. 10.1016/j.biocel.2011.12.015 22230366

[B40] Santos C. X. Stolf B. S. Takemoto P. V. Amanso A. M. Lopes L. R. Souza E. B.. (2009). Protein disulfide isomerase (PDI) associates with NADPH oxidase and is required for phagocytosis of Leishmania chagasi promastigotes by macrophages. J. Leukoc. Biol. 86, 989–998. 10.1189/jlb.0608354 19564574

[B41] Seidman D. Hebert K. S. Truchan H. K. Miller D. P. Tegels B. K. Marconi R. T.. (2015). Essential domains of *Anaplasma phagocytophilum* invasins utilized to infect mammalian host cells. PLoS Pathog. 11:e1004669. 10.1371/journal.ppat.1004669 25658707 PMC4450072

[B42] Seidman D. Ojogun N. Walker N. J. Mastronunzio J. Kahlon A. Hebert K. S.. (2014). *Anaplasma phagocytophilum* surface protein AipA mediates invasion of mammalian host cells. Cell. Microbiol. 16, 1133–1145. 10.1111/cmi.12286 24612118 PMC4115035

[B43] Stantchev T. S. Paciga M. Lankford C. R. Schwartzkopff F. Broder C. C. Clouse K. A. (2012). Cell-type specific requirements for thiol/disulfide exchange during HIV-1 entry and infection. Retrovirology 9:97. 10.1186/1742-4690-9-97 23206338 PMC3526565

[B44] Starkey L. A. West M. D. Barrett A. W. Saucier J. M. O'connor T. P. Paras K. L.. (2013). Prevalence of antibodies to spotted fever group Rickettsia spp. and Ehrlichia spp. in coyotes (Canis latrans) in Oklahoma and Texas, USA. J. Wildl. Dis. 49, 670–673. 10.7589/2012-08-215 23778619

[B45] Troese M. J. Carlyon J. A. (2009). *Anaplasma phagocytophilum* dense-cored organisms mediate cellular adherence through recognition of human P-selectin glycoprotein ligand 1. Infect. Immun. 77, 4018–4027. 10.1128/IAI.00527-09 19596771 PMC2738047

[B46] Truchan H. K. Viebrock L. Cockburn C. L. Ojogun N. Griffin B. P. Wijesinghe D. S.. (2016). *Anaplasma phagocytophilum* Rab10-dependent parasitism of the trans-Golgi network is critical for completion of the infection cycle. Cell. Microbiol. 18, 260–281. 10.1111/cmi.12500 26289115 PMC4891814

[B47] Uehara T. Nakamura T. Yao D. Shi Z. Q. Gu Z. Ma Y.. (2006). S-nitrosylated protein-disulphide isomerase links protein misfolding to neurodegeneration. Nature 441, 513–517. 10.1038/nature04782 16724068

[B48] Viebrock L. Evans S. M. Beyer A. R. Larson C. L. Beare P. A. Ge H.. (2014). Orientia tsutsugamushi ankyrin repeat-containing protein family members are Type 1 secretion system substrates that traffic to the host cell endoplasmic reticulum. Front. Cell. Infect. Microbiol. 4:186. 10.3389/fcimb.2014.00186 25692099 PMC4315096

[B49] Walker D. H. (2005). Ehrlichia under our noses and no one notices. Arch. Virol. Suppl. 19, 147–156. 10.1007/3-211-29981-5_12 16358425

[B50] Walker D. H. Dumler J. S. (1996). Emergence of the ehrlichioses as human health problems. Emerging Infect. Dis. 2, 18–29. 10.3201/eid0201.960102 8903194 PMC2639805

[B51] Wan S.-W. Lin C.-F. Lu Y.-T. Lei H.-Y. Anderson R. Lin Y.-S. (2012). Endothelial cell surface expression of protein disulfide isomerase activates β1 and β3 integrins and facilitates dengue virus infection. J. Cell. Biochem. 113, 1681–1691. 10.1002/jcb.24037 22422622

[B52] Wang Y. Berg E. A. Feng X. Shen L. Smith T. Costello C. E.. (2006). Identification of surface-exposed components of MOMP of *Chlamydia trachomatis* serovar F. Protein Sci. 15, 122–134. 10.1110/ps.051616206 16322562 PMC2242375

[B53] Zai A. Rudd M. A. Scribner A. W. Loscalzo J. (1999). Cell-surface protein disulfide isomerase catalyzes transnitrosation and regulates intracellular transfer of nitric oxide. J. Clin. Invest. 103, 393–399. 10.1172/JCI4890 9927500 PMC407899

[B54] Zhang J. Z. Mcbride J. W. Yu X. J. (2003). L-selectin and E-selectin expressed on monocytes mediating *Ehrlichia chaffeensis* attachment onto host cells. FEMS Microbiol. Lett. 227, 303–309. 10.1016/S0378-1097(03)00696-7 14592723

[B55] Zhang J. Z. Popov V. L. Gao S. Walker D. H. Yu X. J. (2007). The developmental cycle of *Ehrlichia chaffeensis* in vertebrate cells. Cell. Microbiol. 9, 610–618. 10.1111/j.1462-5822.2006.00812.x 16987329

[B56] Zhang N. Shaw A. R. Li N. Chen R. Mak A. Hu X.. (2008). Liquid chromatography electrospray ionization and matrix-assisted laser desorption ionization tandem mass spectrometry for the analysis of lipid raft proteome of monocytes. Anal. Chim. Acta 627, 82–90. 10.1016/j.aca.2008.05.058 18790130

[B57] Zhang T. Kedzierska-Mieszkowska S. Liu H. Cheng C. Ganta R. R. Zolkiewski M. (2013). Aggregate-reactivation activity of the molecular chaperone ClpB from Ehrlichia chaffeensis. PLoS ONE 8:e62454. 10.1371/journal.pone.0062454 23667479 PMC3646808

